# High-Precision Detection of Defects of Tire Texture Through X-ray Imaging Based on Local Inverse Difference Moment Features

**DOI:** 10.3390/s18082524

**Published:** 2018-08-02

**Authors:** Guo Zhao, Shiyin Qin

**Affiliations:** School of Automation Science and Electrical Engineering, Beihang University, Haidian District, Beijing 100191, China

**Keywords:** non-destructive testing, digital X-ray image sensor, defect feature map, defect detection, gray level co-occurrence matrix, local inverse difference moment

## Abstract

Automatic defect detection is an important and challenging issue in the tire industrial quality control. As is well known, the production quality of tire is directly related to the vehicle running safety and passenger security. However, it is difficult to inspect the inner structure of tire on the surface. This paper proposes a high-precision detection of defects of tire texture image obtained by X-ray image sensor for tire non-destructive inspection. In this paper, the feature distribution generated by local inverse difference moment (LIDM) features is proposed to be an effective representation of tire X-ray texture image. Further, the defect feature map (DFM) may be constructed by computing the Hausdorff distance between the LIDM feature distributions of original tire image and each sliding image patch. Moreover, DFM may be enhanced to improve the robustness of defect detection algorithm by a background suppression. Finally, an effective defect detection algorithm is proposed to achieve the pixel-level detection of defects with high precision over the enhanced DFM. In addition, the defect detection algorithm is not only robust to the noise in the background, but also has a more powerful capability of handling different shapes of defects. To validate the performance of our proposed method, two kinds of experiments about the defect feature map and defect detection are conducted to demonstrate its good performance. Moreover, a series of comparative analyses demonstrate that the proposed algorithm can accurately detect the defects and outperforms other algorithms in terms of various quantitative metrics.

## 1. Introduction

Due to unclean production environment and undesired manufacturing facilities used in the tire manufacturing process, tire components may be contaminated by various defects, such as metallic impurities, bubble, and overlap [1]. Moreover, it is difficult to detect the inner structure defect of tire on the surface. Thus, digital X-ray image sensor can be used to build X-ray detector or camera for X-ray non-destructive testing (NDT), which is widely used in quality assurance and development processes, such as integrated circuit packaging inspection [2]. In our work, we applied the X-ray image sensor on NDT in automotive industry. Tires are checked against the structural requirements and safety regulations by using X-ray NDT system to guarantee they comply. Automatic X-ray inspection has long been a desired quality control function in tire manufacturing. It is widely recognized that automatic inspection would promote the needed improvements in the manufacturing craftsmanship and production quality. Although computer vision has been applied to the X-ray inspection system in tire industry, automatic defect inspection and identification is still done by human observers in many tire manufacturing companies [3]. However, the subjective consciousness and physical strength of individuals cannot meet the requirement of modern productions considering the high-intensity and long-time tough work environment.

In our paper, a new analytical approach from the visualization perspective is presented to analyze the arrangement characteristics of co-occurrence values in gray level co-occurrence matrix (GLCM) [4] for the tire X-ray image. In this way, we found that the classical feature extracted from GLCM may be interpreted as the summation of all elements in the matrix, which is the Hadamard product of a weighting matrix and GLCM. Based on the above analysis, the distribution with local inverse difference moment (LIDM) features is presented to be an effective descriptor of tire X-ray texture image. Further, with the LIDM feature distribution, the original tire X-ray image and the image patches obtained by the sliding window approach are analyzed comparatively. The presenting characteristic of defects by LIDM feature distribution shows that the feature distribution of original tire X-ray image is similar to the non-defective image patches. On the contrary, there are contrasting differences on the LIDM feature distributions between original tire X-ray image and the defective image patches. In this way, the defect feature map can be constructed through analyzing the differences of the LIDM feature distributions between original tire X-ray image and each image patch. To improve the robustness and the precision of our proposed defect detection method, the defect feature map is then to be enhanced by our proposed background suppression method. Finally, an effective detection algorithm is proposed to detect the tire X-ray texture defect over the enhanced defect feature map. In addition, the defect detection algorithm is not only robust to the noise in the background, but also has a more powerful capability of handling different shapes of defects.

The remainder of this paper is organized as follows. Section 2 provides the background and related work about the defect detection in many industrial production processes. In Section 3, two classical features based on GLCM are first discussed, and then a new analytical approach from the visualization perspective is presented to analyze the arrangement characteristics of co-occurrence values in GLCM for the tire X-ray image. The computing approach to the LIDM feature distribution is also presented in this section. Section 4 presents the construction method of defect feature map, and then the defect feature map is enhanced by our proposed background suppression. Moreover, our proposed detection method of defects with defect feature map is also given in detail. Section 5 further validates the robustness and effectiveness of the defect detection method by various experiments on a series of real tire X-ray texture images, which are obtained from the non-destructive inspection system. Then, the comparative results through various quantitative metrics are also provided to demonstrate the good performance of our proposed algorithm. In Section 6, the discussion and comments are conducted to expound the influence of parameters in the enhancement algorithm of defect feature map. Finally, the whole paper is concluded in Section 7.

## 2. Background and Related Works

Over the past few decades, many researchers have paid attention to the nondestructive defect defection for industrial products, such as liquid crystal display (LCD) [5], steel [6], and fabric [7,8,9,10,11,12,13,14,15,16,17,18]. In the industrial manufacturing process, visual inspection plays an important role in the quality control. Especially for the fabric quality control, many studies on defect detection were presented to achieve the automatic inspection, and the methods may be mainly categorized into six classes [19]: statistical [7,8,9,10], spectral [11,12], model-based [13], learning [14,15], structural [16] and other approaches [17,18]. In addition, the common statistical approaches include auto-correlation [7], gray level co-occurrence matrix (GLCM) [7,8,9] and fractal method [10]. In general, to improve the robust of defect detection method, the texture image is always represented by combining GLCM with other methods, such as autocorrelation [7], wavelet transform [8] and Gabor transform [9,11,12].

Haralick et al. presented dozens of features based on GLCM, such as contrast, homogeneity, and energy [20]. Typically, various texture features are always calculated based on GLCM to characterize the texture image in industrial manufacturing process. In [7], autocorrelation function was used to determine the pattern period of yarn-dyed fabric, and the GLCM was applied to represent the texture. In addition, it might be difficult to detect the defect in the aperiodic texture image. It is also worth mentioning that the GLCM was not calculated to extract the texture features, but it was regarded as the characteristic matrix of the original image and used to compute the distance between the detected images and the template image [7]. The most obvious in [8] is that the GLCM was not computed from the original texture image, but from the subband decomposed by the wavelet transform method. Jeong-Yun Lee et al. proposed an effective inline inspection method for non-repeating patterns on TFT-LCDs which can be quickly applied to current manufacturing process and easily automated [5].

With the development of artificial intelligence and the enhancement of computer capacity and performance, deep learning methodologies and technologies have been progressing at high speed and have been successfully applied in various fields [21,22,23,24,25,26,27]. In terms of defect detection, Weimer D et al. proposed a novel deep convolutional neural networks (CNNs) architecture to detect defects, which designed a 12-dimensional output to represent 12 different categories [28]. In [29], an elaborate deep CNN model was designed to automatically extract powerful features with less prior knowledge for defect detection. Moreover, the experimental results achieved a high accuracy of 99.8% of correct defect detection rate on the german association for pattern recognition dataset, and outperforming the state-of-the-art methods while at the same time keeps a high processing speed. However, because the generation of training data is expensive, deep learning might not be an appropriate technology [28].

Recently, the literature based on GLCM demonstrates its good performance in many texture analytical tasks [30,31,32,33,34]. In the classical methods based on GLCM, dozens of texture features are calculated from GLCM, and then fused by the feature fusion method [34]. Actually, mainly caused by uncertainty, not every feature can characterize the texture image properly. Thus, we analyze the characteristic of GLCM from the visualization perspective for the tire X-ray image, and present a new texture descriptor based on the LIDM feature distribution instead of various feature values. In this way, especially in the industrial manufacturing process, the tire X-ray texture image may be characterized properly and exactly with the LIDM feature distribution.

## 3. Proposed Texture Descriptor Based on GLCM

### 3.1. Texture Feature Extraction Based on GLCM

The GLCM is a statistical method for representing the spatial correlation of gray levels co-occurred in texture image, and it mainly describes the regularity of distribution at distance and direction of adjacent pixels. However, it is just a statistical technology to calculate the co-occurrence frequency of gray levels. In [20], Haralick et al. proposed fourteen types of features based on GLCM to describe the texture variation.

For an original texture image I:Ω→R with gray level ρ, its corresponding GLCM with orientation angle of θ, $P_{\theta }$, is a square matrix with the same width and height of ρ×ρ. For a fixed distance *d* between the neighboring pixel pair, the various entry of GLCM matrix $P_{\theta }={\left[p_{\theta }^{ij}\right]}_{\rho \times \rho }$ is defined as follows [4]

(1) $$p_{\theta }^{ij}\;=\;\left\{\begin{array}{cc} \sum_{k=1}^{p-1}\sum_{l=0}^{q-1}\delta \left(i-I\left(k,l\right)\right)\delta \left(j-I\left(k+d,l\right)\right), & \theta \;=\;0^{\circ }\\ \sum_{k=1}^{p-1}\sum_{l=0}^{q-1}\delta \left(i-I\left(k,l\right)\right)\delta \left(j-I\left(k+d,l\right)\right), & \theta \;=\;45^{\circ }\\ \sum_{k=1}^{p-1}\sum_{l=0}^{q-1}\delta \left(i-I\left(k,l\right)\right)\delta \left(j-I\left(k+d,l\right)\right), & \theta \;=\;90^{\circ }\\ \sum_{k=1}^{p-1}\sum_{l=0}^{q-1}\delta \left(i-I\left(k,l\right)\right)\delta \left(j-I\left(k+d,l\right)\right), & \theta \;=\;145^{\circ } \end{array}\right.$$

where

(2) $$\delta \left(a-b\right)=\left\{\begin{array}{cc} 1,\;\; & a=b\\ 0,\;\; & a\neq b \end{array}\right.$$

and (k,l) denotes the coordinate of a given image, while I(k,l) denotes the corresponding gray values. The distance *d* in computing GLCM is restricted to one step-size in our work. Based on GLCM, two key representative features can be extracted as below. The first one is named CON (contrast) which reflects the depth and smoothness of image texture structure [35] and is calculated with

(3) $$C\;O\;N=\sum_{i=0}^{T-1}\sum_{j=0}^{T-1}{\left|i-j\right|}^{2}p_{\theta }^{ij}$$

where *T* denotes the width or height of GLCM. The second one is IDM (inverse difference moment), which refers to the uniformity of the image intensity and is a measurement of lack of variability or the amount of local similarity [31]; its definition may be given as

(4) $$I\;D\;M=\sum_{i=0}^{T-1}\sum_{j=0}^{T-1}\frac{p_{\theta }^{ij}}{{1+|i-j|}^{2}}$$

In fact, Haralick et al. proposed fourteen types of feature statistics based on GLCM to describe the texture image in [20]. Only two of them are selected as our support tools to build new analytical method in this paper.

### 3.2. Visualization and Characteristic Analysis of GLCM

In this Section, a new analytical approach from the visualization perspective is proposed to analyze the arrangement characteristic of co-occurrence values in GLCM. Based on the definition of CON, the CON weighting matrix $W_{C\;O\;N}={\left[w_{C\;O\;N}^{ij}\right]}_{\rho \times \rho }$ may be defined with

(5) $$w_{C\;O\;N}^{ij}={\left|i-j\right|}^{2}$$

In this way, the CON calculated in Equation (3) may be redefined by

(6) $$\begin{array}{cc} C\;O\;N & =\sum_{i=0}^{T-1}\sum_{j=0}^{T-1}{\left|i-j\right|}^{2}p_{\theta }^{ij}\\ & =\sum_{i=0}^{T-1}\sum_{j=0}^{T-1}w_{C\;O\;N}^{ij}p_{\theta }^{ij} \end{array}$$

Similarly, the IDM weighting matrix $W_{I\;D\;M}={\left[w_{I\;D\;M}^{ij}\right]}_{\rho \times \rho }$ may be defined by

(7) $$w_{I\;D\;M}^{ij}=\frac{1}{{1+|i-j|}^{2}}$$

Similarly, IDM has a similar expression as Equation (6) and is calculated by

(8) $$\begin{array}{cc} I\;D\;M & =\sum_{i=0}^{T-1}\sum_{j=0}^{T-1}\frac{1}{{1+|i-j|}^{2}}p_{\theta }^{ij}\\ & =\sum_{i=0}^{T-1}\sum_{j=0}^{T-1}w_{I\;D\;M}^{ij}p_{\theta }^{ij} \end{array}$$

**Figure 1:**
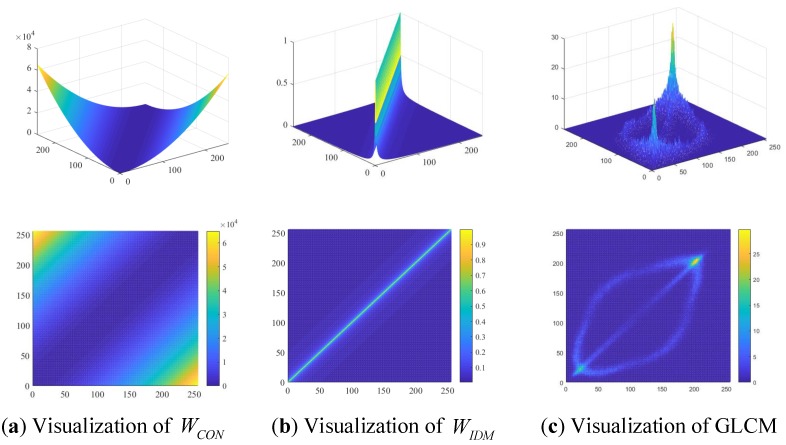
Visualization of weighting matrices $W_{C\;O\;N}$, $W_{I\;D\;M}$ and GLCM from different viewing angles.

To clarify the extraction of contrast from the visualization perspective, the visualization of the CON weighting matrix $W_{C\;O\;N}$, the IDM weighting matrix $W_{I\;D\;M}$ and GLCM $P_{\theta }$ are shown in Figure 1.

It is a remarkable characteristic in Figure 1a that the element value of matrix $W_{C\;O\;N}$ gradually becomes greater with the distance departing from the secondary diagonal. As is defined above in Equations (5) and (7), the IDM weighting matrix $W_{I\;D\;M}$ is actually inverse of the CON weighting matrix $W_{C\;O\;N}$, and the additional 1 in the denominator of $W_{I\;D\;M}$ is mainly to guarantee that the value of the denominator is not zero. Figure 1b shows that the element values of $W_{I\;D\;M}$ are decreasing exponentially with the distance departing from the secondary diagonal. Furthermore, we can define the CON feature matrix ${\tilde{W}}_{C\;O\;N}={\left[{\tilde{w}}_{C\;O\;N}^{ij}\right]}_{\rho \times \rho }$ with

(9) $${\tilde{w}}_{C\;O\;N}^{ij}=w_{C\;O\;N}^{ij}p_{\theta }^{ij}$$

In this way, the CON feature matrix ${\tilde{W}}_{C\;O\;N}$ may be calculated through matrix multiplication by

(10) $${\tilde{W}}_{C\;O\;N}=W_{C\;O\;N}\circ P_{\theta }$$

where the operator “∘” denotes the Hadamard product, which produces another matrix where each element is the product of elements of the original two matrices at the same position. It is noteworthy that the Hadamard product is simply entrywise multiplication. By sharing a similar mathematical expression with Equation (10), the IDM feature matrix ${\tilde{W}}_{I\;D\;M}$ may be expressed in the following form:

(11) $${\tilde{W}}_{I\;D\;M}=W_{I\;D\;M}\circ P_{\theta }$$

It is worth noticing that the weighting matrices $W_{C\;O\;N}$, $W_{I\;D\;M}$ and GLCM $P_{\theta }$ must have the same size, and necessarily be square matrices.

**Figure 2:**
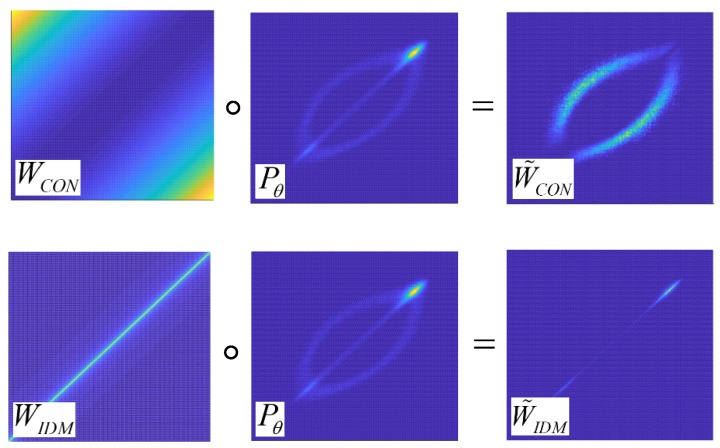
Illustration of visualization about enhancement of CON and IDM.

Unlike the classical calculating process of feature extraction, we present a new analysis method through matrix multiplication to explain the extracting process of texture features. As illustrated in Figure 2, the computing approach to matrix ${\tilde{W}}_{C\;O\;N}$ might be interpreted as the Hadamard product of the GLCM and the weighting matrix $W_{C\;O\;N}$. Similarly, the matrix ${\tilde{W}}_{I\;D\;M}$ may also be obtained by the Hadamard product of the GLCM and the weighting matrix $W_{I\;D\;M}$. By the definition of CON and IDM, both are actually the sum of all the element values in ${\tilde{W}}_{C\;O\;N}$ and ${\tilde{W}}_{I\;D\;M}$, respectively.

To simplify the feature extracting process, CON may be calculated in a more concise vector form. More specifically, the matrices $W_{C\;O\;N}$ and $P_{\theta }$ may be vectorized as $W_{C\;O\;N}$ and $P_{\theta }$, and then the CON can be calculated by an inner product between vector $W_{C\;O\;N}\in R^{(256\times 256)\times 1}$ and $P_{\theta }\in R^{(256\times 256)\times 1}$ as below

(12) $$C\;O\;N=\langle W_{C\;O\;N},P_{\theta }\rangle$$

In addition, the computation of IDM shares a similar mathematical expression, the matrix $W_{I\;D\;M}$ may also be vectorized as $W_{I\;D\;M}\in R^{(256\times 256)\times 1}$, and then the IDM feature is calculated by

(13) $$I\;D\;M=\langle W_{I\;D\;M},P_{\theta }\rangle$$

Based on the analysis of approach to the feature extraction by our proposed analytical method, it is important to construct an effective and reasonable weighting matrix for extracting proper texture features. With different weighting matrices, various features may be extracted to represent the attributes of texture structure. In this way, a series of effective weighting matrices may play an important role in the extraction of texture features.

### 3.3. Texture Descriptor of Tire X-ray Image Based on LIDM Feature

**Figure 3:**
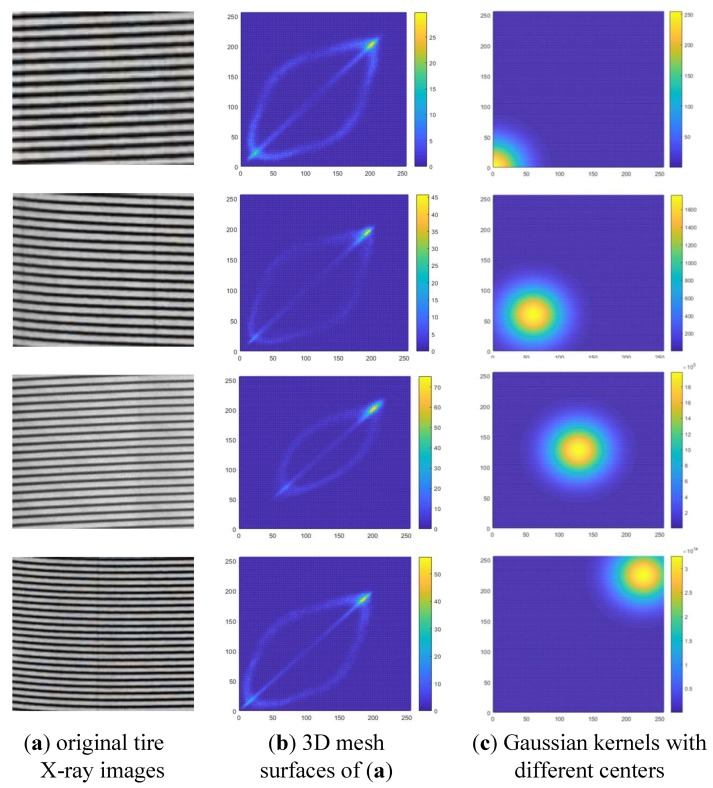
Exhibition of characteristic analysis of different tire X-ray images.

As mentioned above, it is crucial to construct an effective weighting matrix for extracting the effective feature from GLCM. Figure 3a shows several tire X-ray images, which were obtained from the tire non-destructive defect inspection system. To illustrate the distribution regularity of the elements in GLCM, we calculate the average of GLCMs ${\tilde{P}}_{\theta }={\left[{\tilde{p}}_{\theta }^{ij}\right]}_{\rho \times \rho }$ at four angles defined in Equation (1) by

(14) $${\tilde{p}}_{\theta }^{ij}=\frac{1}{4}\sum_{l=1}^{4}k_{l}p_{\theta }^{ij}$$

where $k_{l}$ is the weighting parameter, which is set to be 1 for illustration here. However, in the following extraction procedure of LIDM feature distribution, the weighting parameters are chosen empirically, and more discussion is provided in Section 5.1. The average GLCMs of tire X-ray images are visualized and illustrated in Figure 3b. Moreover, Figure 3 shows that the tire X-ray images have a similar characteristic and the elements in GLCM always distribute around the secondary diagonal.

Based on the analysis of features extraction shown in Figure 3, we may construct a proper Gaussian kernel, which takes the place of the classical weighting matrix, such as $W_{C\;O\;N}$ and $W_{I\;D\;M}$. Figure 3c illustrates the visualizations of various Gaussian kernels, of which the centers vary at the secondary diagonal. Note that, because of the constraint condition of Hadamard product, the Gaussian kernels should have the same size with GLCM. In this way, in order to better represent tire X-ray texture image, the Gaussian kernel is designed to be a weighting matrix in our study by

(15) $$G_{\sigma ,\mu }\left(x\right)=\frac{1}{\sqrt{2\pi }\sigma }e^{-\frac{{∥x-\mu ∥}^{2}}{2\sigma^{2}}}$$

where σ and μ denote the deviation and center of Gaussian kernel, respectively. Moreover, the center of Gaussian kernel μ locates at the secondary diagonal in our work, and the visualizations of different Gaussian kernels are illustrated in Figure 3c. In this way, the Gaussian kernel may be constructed and applied to extract a feature value, which is called LIDM feature value. According to Equations (12) and (13), for a fixed center $\mu^{*}$ of Gaussian kernel, the LIDM feature may be calculated from the following formula

(16) $$\begin{array}{cc} L\;I\;D\;M_{\sigma }\left(\mu^{*}\right) & =\langle \sum_{i=1}^{4}k_{i}P_{\theta_{i}},G_{\sigma ,\mu^{*}}\rangle \\ & ={\left(\sum_{i=1}^{4}k_{i}P_{\theta_{i}}\right)}^{T}G_{\sigma ,\mu^{*}} \end{array}$$

where $\mu^{*}$ denotes any center located at the secondary diagonal and G is the vectorization of Gaussian kernel *G*. In this way, various Gaussian kernels may be constructed with different centers at the secondary diagonal, and a series of LIDM features may be computed by Equation (16). Therefore, for better charactering tire X-ray texture image, we propose the LIDM feature distribution generated by these LIDM feature values to be a new texture descriptor instead of dozens of classical features.

**Figure 4:**
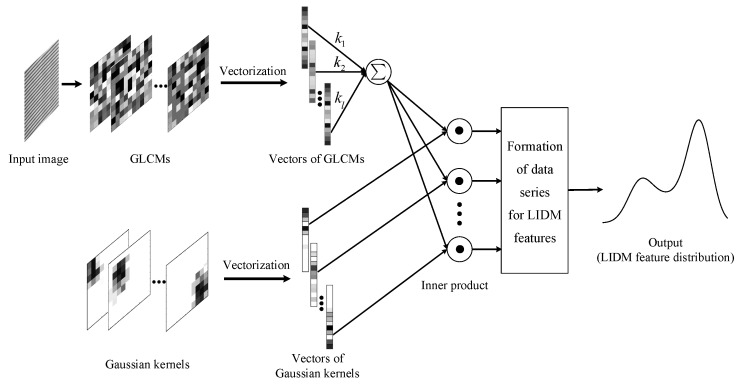
The extraction procedure of LIDM feature distribution.

As shown in Figure 4, the whole extraction procedure of LIDM feature distribution consists of two parallel channels and a weighted fusion unit. In the first channel, for an input tire X-ray image χ, four GLCMs $P_{\theta_{i}},\theta_{i}\in \left\{0^{\circ },45^{\circ },90^{\circ },145^{\circ }\right\}$ are calculated and vectorized as $P_{\theta_{i}},i=1,2,3,4$. Then, the four vectors of GLCMs are fused by the weighting parameter $k_{i}$, the setting of which is discussed in Section 5.1. Meanwhile, in the second channel, a series of Gaussian kernels $G_{\sigma ,\mu }$ may be constructed with different centers and vectorized as $G_{\sigma ,\mu }$ to generate the LIDM feature distribution subsequently. In this way, the LIDM feature distribution of input tire image χ is generated and output with formula

(17) $$\begin{array}{cc} L\;I\;D\;M_{\sigma }^{\chi } & =\langle \sum_{i=1}^{4}k_{i}P_{\theta_{i}},G_{\sigma ,\mu }\rangle \\ & ={\left(\sum_{i=1}^{4}k_{i}P_{\theta_{i}}\right)}^{T}G_{\sigma ,\mu } \end{array}$$

where μ ranges from the center of ${\left(0,0\right)}^{T}$ to ${\left(255,255\right)}^{T}$ successively at the diagonal of Gaussian kernel, since the size of Gaussian kernel is restricted to 256×256 for gray image in our work. By taking different values of the parameter μ, various Gaussian kernels may be constructed and applied to calculate a group of LIDM feature values. In this way, an effective texture descriptor by the LIDM feature distribution, generated by the group of LIDM feature values, is proposed to characterize the tire X-ray image.

### 3.4. Representing Characteristics of Defects in Proposed Texture Descriptor

In the classical feature extraction method of texture image based on GLCM, dozens of features may be calculated to characterize a texture image. However, these features always need to be fused by a weight fusion algorithm, or need a dimensional reduction algorithm to solve the high-dimension problem [34]. In our work, the LIDM feature distribution instead of dozens of features is presented to be a new texture descriptor.

**Figure 5:**
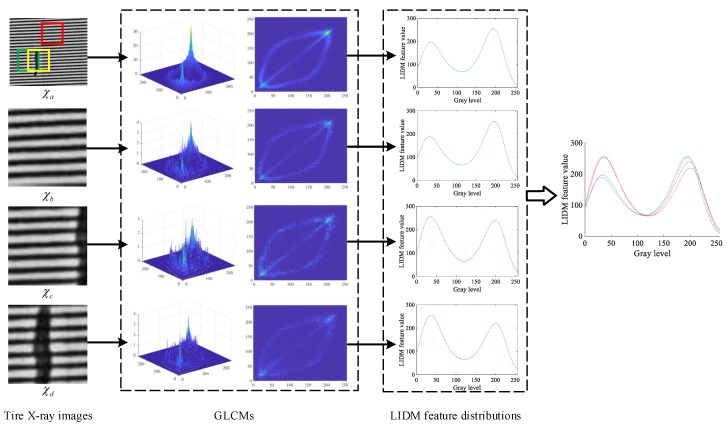
Comparison of characteristics of defects by LIDM feature distribution.

It is noteworthy that the LIDM feature distribution shows a discriminative characteristic on the non-defective and defective texture image. As shown in Figure 5, $\chi_{a}$ is the original tire X-ray image, and the boxes with different colors were selected to validate the discriminative characteristic. Here, the red, green and yellow regions in image $\chi_{a}$ correspond to images $\chi_{b}$, $\chi_{c}$ and $\chi_{d}$, respectively. In Figure 5, we found that the LIDM feature distributions of $\chi_{a}$ and $\chi_{b}$ have a high similarity. Meanwhile, the similarity is also shown in Rows 3 and 4, and similar LIDM feature distributions are marked by the same color for clarity. Although the original tire X-ray image are actually flawed, it has a similar feature distribution with the non-defective tire X-ray image $\chi_{b}$. Since the defect makes up only a small portion of the whole texture image, the defect region has a less influence on the changing of statistics of co-occurrence gray levels. In this way, the sliding window method may be applied to extract the texture image patches, which are then used to generate the LIDM feature distributions. Moreover, the LIDM feature distribution generated from the original tire X-ray image may be set to be a root distribution, which can be used to measure whether the image patch is a defective image.

## 4. Detection Algorithm of Tire Texture Defects

### 4.1. Construction of Defect Feature Map Based on Texture Descriptor

Because of the lower influence on the changing of statistics of co-occurrence gray levels by the defect in the original tire X-ray image, the LIDM feature distribution of original tire image shows a similarity with that of non-defective image patches. In this section, to measure the degree of difference between different LIDM feature distributions in a quantitative method, the Hausdorff distance is used to compute the degree of difference in our work.

**Definition** **1.** *Given two finite two point sets $A=\{a_{1},a_{2},\ldots ,a_{p}\}$ and $B=\{b_{1},b_{2},\ldots ,b_{q}\}$, the bidirectional Hausdorff distance is defined as*

(18) $$H\left(A,B\right)=\max\left(h(A,B),h(B,A)\right)$$

*where h(A,B) is the one-side Hausdorff distance, and is defined by*

(19) $$h\left(A,B\right)=\max_{a\in A}\min_{b\in B}\left\{d(a,b)\right\}$$

*and d(a,b) denotes the distance between any a∈A and any b∈B, which may be defined as Euclidean distance or others.*

The Hausdorff distance is a measure of how much two nonempty sets *A* and *B* in a metric space resemble each other with respect to their positions. It should be noted that the Hausdorff distance is oriented, which means that h(A,B) is usually not equal to h(B,A). In our work, the bidirectional Hausdorff distance is applied to measure the difference between the LIDM feature distributions, and the Euclidean norm is selected to be the distance d(A,B).

As shown in Table 1, $L\;I\;D\;M^{\chi_{a}}-L\;I\;D\;M^{\chi_{d}}$ denote the LIDM feature distributions that are generated from the corresponding tire X-ray images labeled by $\chi_{a}-\chi_{d}$ in Figure 5. Based on the discussion on the differences between the LIDM feature distributions in Section 3.4, the Hausdorff distance between $L\;I\;D\;M^{\chi_{a}}$ and $L\;I\;D\;M^{\chi_{b}}$ is approximate to the distance between $L\;I\;D\;M^{\chi_{c}}$ and $L\;I\;D\;M^{\chi_{d}}$. On the contrary, the Hausdorff distances between $L\;I\;D\;M^{\chi_{b}}$ and $L\;I\;D\;M^{\chi_{c}}$, $L\;I\;D\;M^{\chi_{b}}$ and $L\;I\;D\;M^{\chi_{d}}$ show that there is a significant discrimination between the non-defective and defective tire X-ray image. From the perspective of root distribution, which is the specific LIDM feature distribution generated from the original tire X-ray image, the defective image patches differ more from the original image than non-defective image patches.

**Figure 6:**
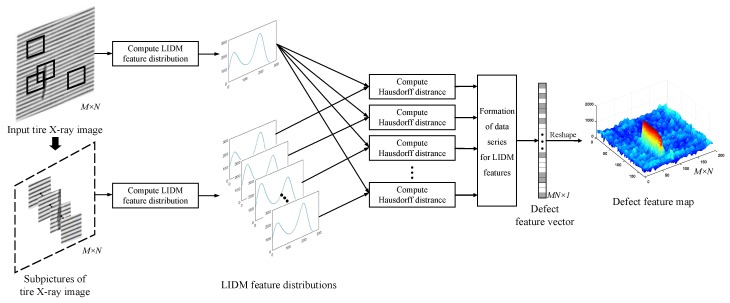
Construction of defect feature map.

In this way, as shown in Figure 6, the Defect Feature Map (DFM) of tire X-ray image may be constructed based on Hausdorff distance by the following four steps.

In the first step, subpictures of tire X-ray image may be obtained from the input tire X-ray image $\chi^{*}$ with the size M×N based on the sliding window method, and the number of subpictures equals to MN.

In the second step, there are two branches that the LIDM feature distributions of the input image $\chi^{*}$ and its subpictures $\chi_{s}\left(s=1,2,\ldots ,MN\right)$ may be computed, separately.

In the third step, we mark the LIDM distribution $L\;I\;D\;M^{\chi^{*}}$ computed from $\chi^{*}$ as the root distribution. Given the two distributions $L\;I\;D\;M^{\chi^{*}}=\left\{\alpha_{0},\alpha_{1},\ldots ,\alpha_{255}\right\}$ and $L\;I\;D\;M^{\chi_{s}}=\left\{\beta_{0},\beta_{1},\ldots ,\beta_{255}\right\}$, the bidirectional Hausdorff distance D(s) may be calculated by

(20) $$\begin{array}{cc} D(s) & =H(L\;I\;D\;M^{\chi^{*}},L\;I\;D\;M^{\chi_{s}})\\ & =\max\left(h\left(L\;I\;D\;M^{\chi^{*}},L\;I\;D\;M^{\chi_{s}}\right),h\left(L\;I\;D\;M^{\chi_{s}},L\;I\;D\;M^{\chi^{*}}\right)\right) \end{array}$$

where

(21) $$\begin{array}{c} h\left(L\;I\;D\;M^{\chi^{*}},L\;I\;D\;M^{\chi_{s}}\right)=\max_{\alpha \in L\;I\;D\;M^{\chi^{*}}}\min_{\beta \in L\;I\;D\;M^{\chi_{s}}}\left\{∥\alpha ,\beta {∥}^{2}\right\} \end{array}$$

(22) $$\begin{array}{c} h\left(L\;I\;D\;M^{\chi_{s}},L\;I\;D\;M^{\chi^{*}}\right)=\max_{\beta \in L\;I\;D\;M^{\chi_{s}}}\min_{\alpha \in L\;I\;D\;M^{\chi^{*}}}\left\{∥\beta ,\alpha {∥}^{2}\right\} \end{array}$$

In the fourth step, since the dimension of Hausdorff distance vector *D* is equal to MN×1, we may reshape the Hausdorff distance vector *D* with MN×1 to the DFM with M×N.

### 4.2. Enhancement of Defect Features Based on Background Suppression

**Figure 7:**
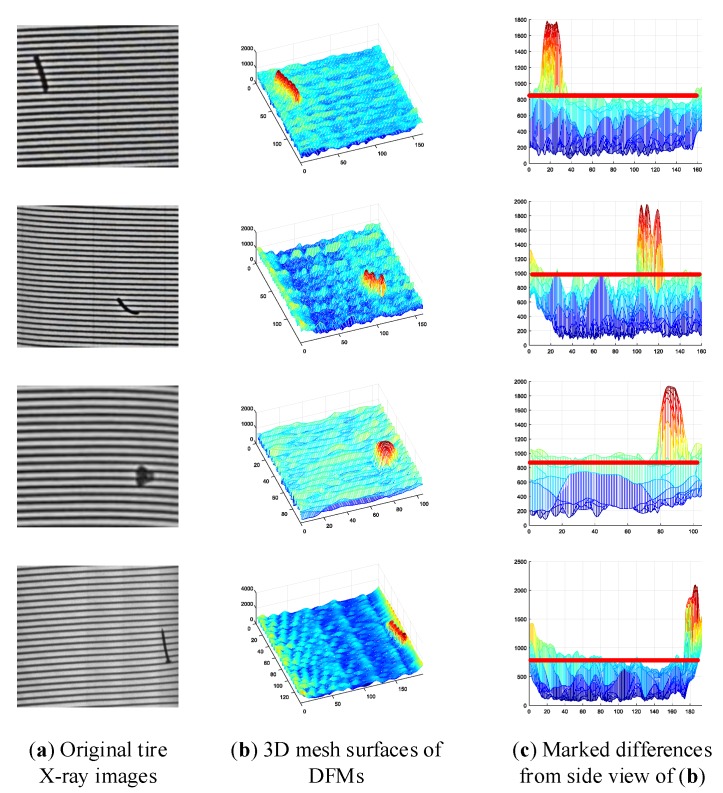
Illustration of DFMs from different tire X-ray images.

As shown in Figure 7, the defects in the DFMs show an evident discrimination with the background. Although many experiments conducted on the tire X-ray image database validate the feasibility of our proposed construction algorithm of DFM, there are still numerous interference points affecting the detection precision. Fortunately, it may be known through analysis of DFMs that three specific characteristics are helpful to eliminate the affection of interference points, which may be listed as follows

(1) The feature values of defects are always much greater than that of background, except that some feature values in the background are great enough to affect the performance of detection algorithm.
(2) The feature values in the background always fluctuate in a large range, but most of these feature values are distributed below a certain level, which is marked by the red line in Figure 7.
(3) The defect region makes up only a small portion of the whole tire X-ray image, on the contrary, the background takes up most of the tire X-ray image.

Based on above major advantages, the DFM may be enhanced by the background suppression method in our work and the enhanced defect feature map (EDFM) $\tilde{D}\left(s\right)$ may be computed by

(23) $$\tilde{D}\left(s\right)=\sum_{k=1}^{M\;N}|D^{p}\left(s\right)-D^{p}\left(k\right)|$$

where *D* is the basic DFM; *M* and *N* are the width and height of DFM, respectively; and p(p=1,2,3,…) is the background suppression index.

For the large disparity in the proportion of defect and background regions, the background may be weakened by our proposed background suppression method. For each value D(s) at point s in DFM, the differences between it and all other points are first calculated, and then the current value D(s) may be updated with the summation of all the differences. Meanwhile, the discrimination between the defect and background regions may be preserved or even improved. It is worth discussing that the parameter *p* determines the performance of background suppression method. The feature values in background approach zero as *p* being greater, and more discussion about the background suppression index was provided in Section 6.

### 4.3. Detection Algorithm of Defects with Defect Feature Map

To achieve the pixel-level defect detection result, a high-precision detection algorithm of defects with DFM is proposed in this section. It is indisputable that the texture of a point is undefined, and texture has a contextual property, of which the definition must involve gray values in a spatial neighborhood. Thus, the feature value in the DFM is computed from each sliding image patch, but the edge of DFM are actually not the real edge of defect.

**Figure 8:**
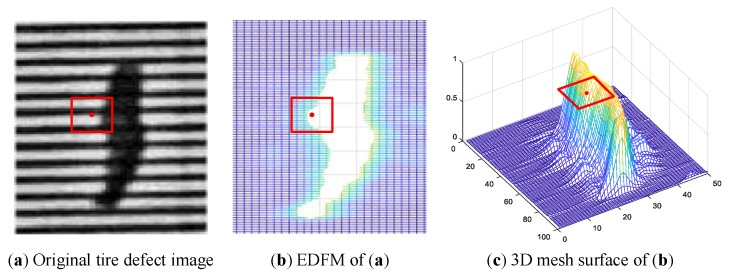
Exhibition of difference between defect image and DFM.

The feature value at the red point in DFM is computed from the subpicture marked by the red rectangle in Figure 8. Figure 8b shows that the red point locates at the inside of defect region, however, the red point actually belongs to the background, which can be clearly identified in Figure 8a. The reason for this phenomena is that the defect will cover a larger proportion of the sliding image patch with the moving of sliding window. In addition, Figure 8c shows that the features of DFM are becoming greater when the defect region gradually enters the sliding window.

By the enlightenment of construction process of DFM, we propose a threshold based sliding window detection (TSWD) algorithm to achieve pixel-level detection of defects. In addition, the defect detection algorithm is not only robust to the noise in the background, but also has a more powerful capability of handling different shapes of defects. Algorithm 1 depicts the steps.

**Algorithm 1** Detection algorithm of defects of tire X-ray image**Input:** Original tire X-ray image *I*, defect feature map $\tilde{D}$ **Output:** Mask result $\tilde{I}$ of defect detection algorithm **Method:**1:Parameters initialization: initialize the size of sliding window to ω×ω, the statistical threshold ξ, the detection threshold δ;2:**while** each point s← 1 to size($\tilde{D}$) **do**3: Obtain the current sliding image patch ${\tilde{\chi }}_{s}$ with size ω×ω in $\tilde{D}$;4: Calculate the number $N_{s}$ of feature values that are greater than the statistical threshold ξ in ${\tilde{\chi }}_{s}$;5: Compute the ratio of $N_{s}$ to the total number of pixels in the current patch ${\tilde{\chi }}_{s}$ by $Ratio=\frac{N_{s}}{\omega^{2}}$;6: **if** Ratio≥δ **then**7:  $\tilde{I}\left(s\right)=1$;8: **else**9:  $\tilde{I}\left(s\right)=0$;10: **end if**11:**end while**

In our proposed detection algorithm of tire X-ray defect, the sliding window method is applied again, and the size of sliding window is the same as that used in the procedure of construction of DFM. Firstly, for the image patch with center point s, the number $N_{s}$ of feature values that are greater than the statistical threshold ξ is counted to ensure that the defect is inside the current image patch. Secondly, the ratio of $N_{s}$ to the whole image patch is computed and then is used to determine whether the current point s belongs to defect. Notice that the parameters of the statistical threshold and the detection threshold are empirically chosen in our work.

### 4.4. Performance Analysis and Comments

Recently, deep learning approaches have achieved tremendous success in various fields, such as segmentation, detection and classification. Certainly, in the industrial manufacture, many studies have put emphasis on the quality visual inspection system with deep learning. In [29], an elaborately designed deep convolutional neural network is employed to automatically extract effective features with less prior knowledge for defect detection, which is also robust to noise.

**Figure 9:**
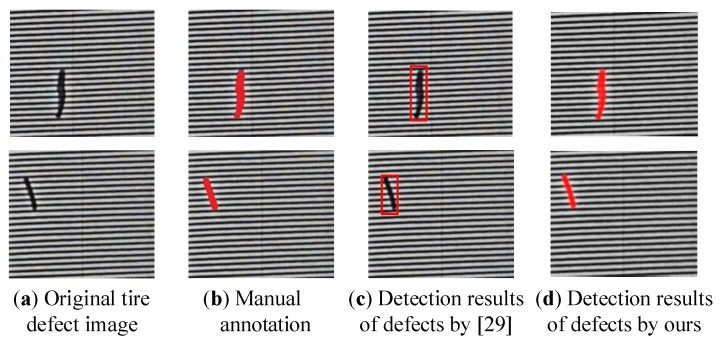
Experimental comparison of different detection algorithms.

As shown in Figure 9, the detection result by [29] demonstrates its good performance, and the defect is detected by the red rectangle with a high accuracy. However, in the tire industrial X-ray inspection system, the ultimate goal is not only to identify whether the tire is acceptable, but also identify to which degree the defect should belong. Therefore, it needs a powerful detection algorithm to achieve the pixel-level detection of defects, and the quality degree of tire can be determined by its size, length and shape based on the pixel-level detection results of defects. Depending on the manufacturing requirement, the automatic detection algorithm of tire defects needs to achieve a pixel-level detection result. In comparison, the defects, marked by red color in Figure 9d, can be detected with pixel-level based on our proposed defect detection algorithm. Moreover, it is obvious that the detection results are very close to the manual annotation. Rather than detecting the defects by rectangles, the detection results in pixel-level could be beneficial to determine the flaw degree of tires.

## 5. Experimental Results and Assessment

### 5.1. Experiment Scheme and Parameters Setting

ITwo groups of experiments were performed to evaluate the performance of our proposed defect detection method of tire X-ray texture image. Several real tire X-ray images from the actual tire industrial X-ray inspection system were collected. The experimental scheme of tire X-ray images included two main parts: experiment towards construction of DFM/EDFM and experiments towards defect detection. In addition, series of experiments with DFM were performed to compare with our proposed detection algorithm. Based on the construction of DFM and EDFM, two famous active contour models, ACWE (Active Contour Without Edge) [36] and LBFACM (Local Binary Fitting based Active Contour Model) [37], are used to implement the segmentation of defects.

**Figure 10:**
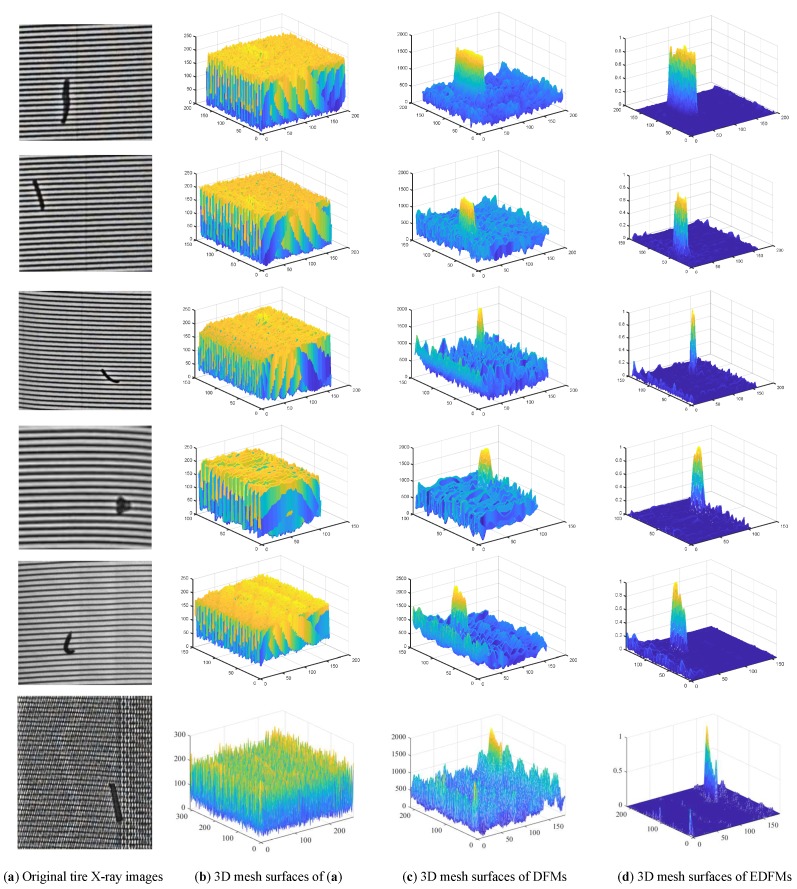
Construction of DFM and EDFM.

The parameters in the proposed algorithm were chosen empirically. In Section 3.1, we have stated that the distance is restricted to one step-size in vertical or horizontal direction in computing GLCM. In addition, in the procedure of LIDM feature extraction, the weighting parameters determine the fusion performance of the GLCMs, and these weighting parameters $k_{i}\left(i=1,2,3,4\right)$ were chosen empirically based on the characteristic of texture. As shown in Figure 10a, the texture is characterized by many horizontal lines, so we chose the weighting parameters at $0^{\circ }$ and $90^{\circ }$ to be slightly greater than at other angles. Besides, there are no evident directional lines in Figure 10a, thus the weighting parameters could be set to the same values.

### 5.2. Experimental Results and Comparative Analysis

#### 5.2.1. Experiment Towards Construction of Defect Feature Map

In this Section, the construction results of DFM and EDFM for different tire X-ray images are provided. A illustrated in Figure 10a, the experiments on several representative images are conducted to validate the performance of feature map construction. There are three strip foreign objects with different size in Rows 1–3, unlike the blob object shown in Row 4. Meanwhile, to validate the robustness of our detection algorithm, the tire X-ray image with the bending foreign object was also selected in our experiments. Furthermore, the tire X-ray image with a much more complex background was added to demonstrate the good performance of our proposed defect detection algorithm.

Figure 10c shows that the defects have become more evident than the background in DFM. However, the features in the background change so dramatically that many interference points may affect the detection precision seriously. Therefore, it is necessary to suppress the background noise while maintaining or even enhancing the defects. Comparing Figure 10c,d, the background in Figure 10d is much smoother and evens than in Figure 10c. Although there are still some interference points in the background, they have almost no influence over the detection precision by our proposed detection algorithm. Notice that the background suppression index was chosen empirically, and its detailed discussion is provided in Section 6.

#### 5.2.2. Experiment Towards Detection Performance of Defects

We conducted two groups of experiments to validate the enhancement performance of our proposed defect enhancement method. Moreover, in each group of experiment, the active contour models (ACWE and LBFACM) were applied to achieve the segmentation of defect in the pixel-level, and the parameters were well tuned.

As shown in Figure 10c,d, it is obvious that the DFM/EDFM consists of two classes of features, the obvious upward bulge denotes the defect region, and the other belongs to the background. The most important is that the features distribution in DFM/EDFM meets the assumption of piecewise constant models that the image intensities are statistically homogeneous in each region. The active contour models show a significant performance for segmenting two-phase gray image, and many studies demonstrate its good performance. Among the many active contour models, ACWE and LBFACM are two representative models that can be applied to the segmentation of defects in the DFM and EDFM. ACWE aims to identify each region of interest by using statistical information inside and outside of evolving curves. As an important improved model based on ACWE, LBFACM utilizes local image information for accurate recovery of desired object boundary. The detail analysis of the comparative experiments is provided in the next section.

#### 5.2.3. Comparative Analysis and Remarks

**Figure 11:**
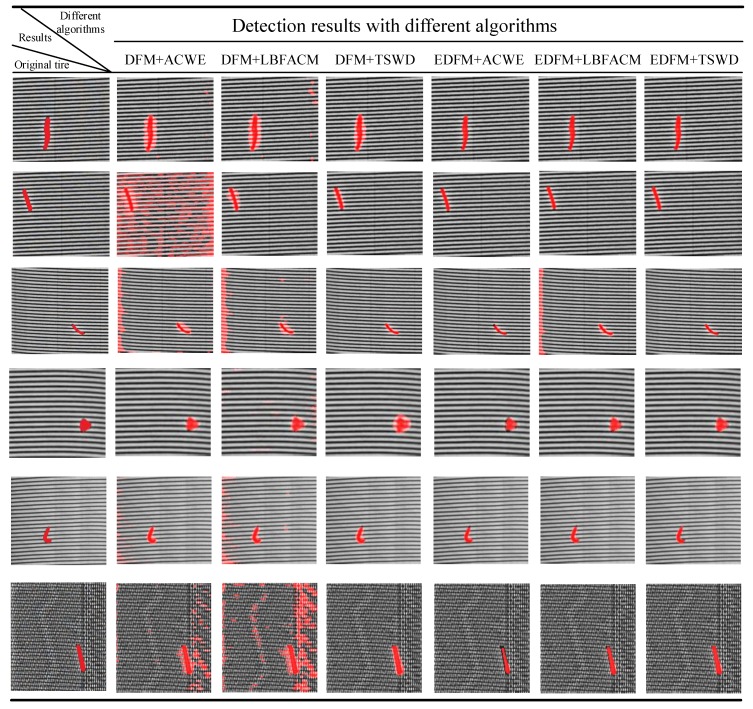
Detection results with different algorithms for tire X-ray images.

As is shown in Columns 2 and 3 of Figure 11, since the features in the background always fluctuate in a relative large range, the ACWE with DFM is difficult to deal with deal with this characteristic of wide fluctuation. Then, the results show bad performance of defect detection, especially for the result in Row 2 where many regions in the background are wrongly detected as the defect. However, for utilizing the local feature information, the result by LBFACM in Row 2 with DFM shows a more powerful ability to detect the defect with a high precision than that by ACWE. Otherwise, unlike the global fluctuation shown in Row 2 of Figure 10c, there are several abnormal local regions that are significantly higher than the background in Row 6 of Figure 10c. Thus, these abnormal local regions may be wrongly detected by LBFACM, and Row 6 in Figure 11 with DFM demonstrate that the performance of defect detection by LBFACM is even a bit worse than that by ACWE. However, comparing with the detection results by classical ACMs, the result shows that the performance of defect detection by oursis much better than that of ACMs with DFM, and validates the high precision and the robustness of our proposed detection algorithm.

Since the features in the background always fluctuate in a large range, the algorithms of defect detection over DFM shown in Columns 2–4 of Figure 11 may not obtain better results. However, it can be seen in the last three columns of Figure 11 that the defects can be detected with a higher precision over EDFM than DFM. The good performance may all be the contribution of the powerful ability of the background suppression method, which is proposed in Section 4.2. Moreover, the results in the last three columns of Figure 11 show that the ACMs are less robust than our proposed method in dealing with the edges of defects.

To analyze the time cost of defect detection over the feature map, all experiments were conducted on a laptop with 4-GB memory and 2.6-GHz intel core Duo processor i5-4210, and the code was implemented by MATLAB R2017a software. For ACMs, the initial evolving contour was set to be close to the defect regions manually, and then the convergence rate can be significantly improved. As shown in Table 2, image 1–6 correspond to the images in row 1–6 of Figure 11. Due to the fluctuation of large range in the feature map, it always needs more time to complete the iteration in ACMs. However, the time cost of our proposed TSWD algorithm was determined by the image size and the computer capacity. In addition, Table 2 validates the analysis that all the detection times in our methods were around 1 s, and they are marked in bold. Furthermore, the detection time may be further reduced by multithreading approach.

### 5.3. Evaluation Metrics and Performance Assessment

**Figure 12:**
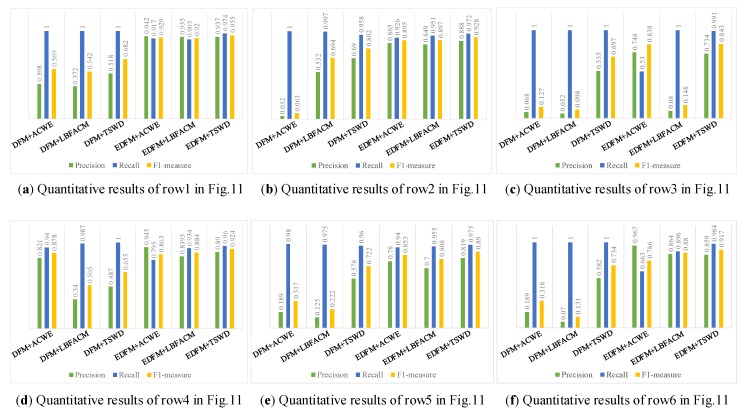
Accuracy comparison of different competing methods.

We conducted experiments using a quantitative metric to compare the performance of the three methods over DFM and EDFM, and the results are presented in Figure 12. For each method, *Precision*, *Recall* and *F1-measure* are chosen to be the quantitative metrics to evaluate their performance. *Precision* is defined as *TP/(TP+FP)*, where *TP* and *FP* are the numbers of pixels that are rightly and wrongly detected, respectively. *Recall* is defined as *TP/(TP+FN)*, where *FN* is the number of right pixels that are not detected. Then, the *F1-measure* is calculated as follows:

(24) $$F1\;-\;measure=\frac{2\times Precision\times Recall}{Precision+Recall}$$

Actually, the *F1-measure* is close to the smaller one of *Precision* and *Recall*. As shown in Figure 12, especially in Figure 12c, the *F1-measure* is much closer to the *Precision* while the *Recall* reaches the highest score. The *Recall* reaches the highest score while the *Precision* is an extremely low score means that too many regions in the background have been wrongly detected when the whole defect has been detected. Thus, the *F1-measure* may be a better quantitative metric to validate the performance of the competing methods. As shown in Figure 12, it is obvious that our proposed defect detection method achieves a good result in the tire X-ray images. In addition, the *F1-measure* demonstrates the better performance of defect detection method proposed by ours than by other methods.

For clarity, the results are also shown in Table 3, where *P*, *R* and *F* denote *Precision*, *Recall* and *F1-measure*, respectively. Moreover, the best results of *F1-measure* of each tire X-ray image are marked in bold. As shown in Table 3, we did not obtain the highest rate on *Precision* and *Recall* in all figures, but all the rates of *F1-measure* obtained by our method are higher than that obtained by other methods.

### 5.4. Comparative Experiments with State-of-the-Art Methods

**Figure 13:**
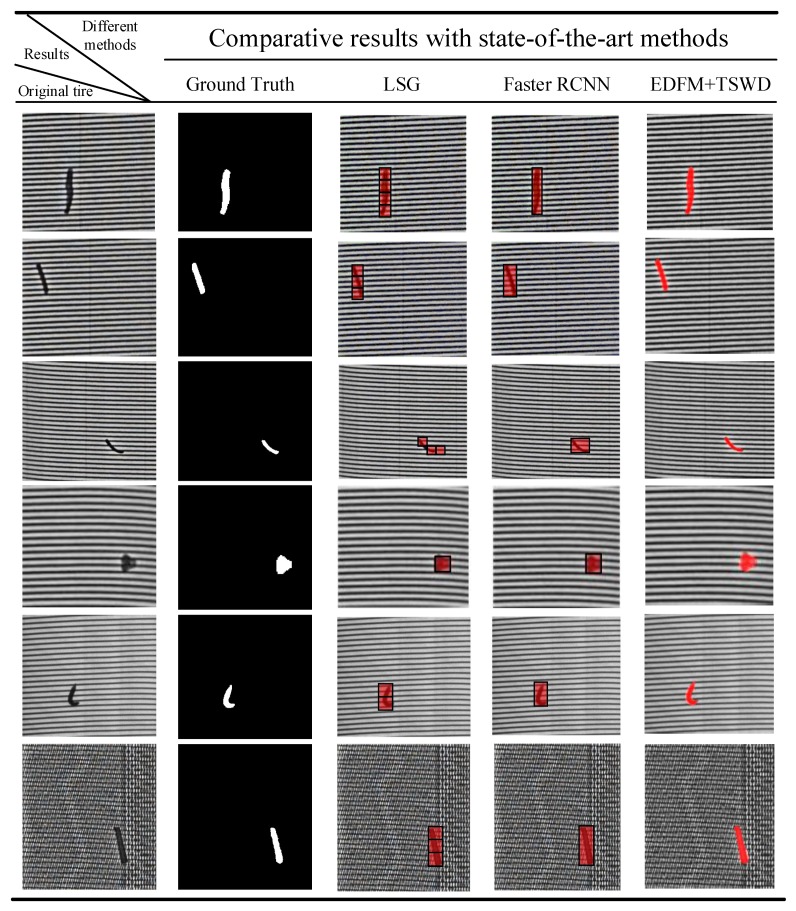
Comparative experiments with state-of-the-art methods.

Two state-of-the-art methods were implemented for comparison, i.e., LSG (lattice segmentation assisted by Gabor filters) [38] and faster RCNN [25]. The LSG method was designed for box- and star-patterned fabric databases, which were provided by Industrial Automation Research Laboratory from Dept. of Electrical and Electronic Engineering of Hong Kong University [38]. However, there are no fixed patterned texture primitive in tire X-ray defect images. To conduct the comparative experiments with the LSG method, the lattice was set to fixed size determined by each tire X-ray image manually. As shown in Figure 13, the defective lattices were marked by red color with different size. In fact, these tire X-ray defective images needed some preprocessing approaches, such as stretch and smooth, to obtain better results. Column 4 of Figure 13 illustrates the detection of defects by faster RCNN, which is an excellent detection method based on CNN framework. Since the tire X-ray image database was relatively small, the data augmentation approach was applied to avoid the overfitting in the training.

As shown in Figure 13, even though the two state-of-the-art methods can detect the defects with a high accuracy, the defects are just detected by rectangles. However, the defects can be detected in pixel-level by our proposed detection method. To evaluate the results with the ground truth images, the defection results were marked in binary images. Since the detection results by LSG and faster RCNN were marked by rectangles, the pixels in the rectangles were set to one, and other pixels were set to zero. Moreover, the definition of evaluation metrics can be found in Section 5.3.

In Table 4, it can be clearly seen that *Recall* can alway reach 100% by LSG and faster RCNN, because the whole defects are detected. However, since the defects have a variety of shapes, the detection rectangles not only mark the defect regions, but also contain the background. Hence, *F1-measure* of the results by LSG and faster RCNN may be at a relative low level. In contrast, we can obtain the highest scores on *F1-measure* in all images, and the best results are marked in bold.

## 6. Discussion and Comments

In our study, the defect enhancement method based on the background suppression has a great influence on the performance of defect detection; the experimental results also validate that the detection results by different methods on EDFM is much better than that on DFM. The defect enhancement method can inhibit background noise significantly while guaranteeing the edge and shape of the defect.

**Figure 14:**
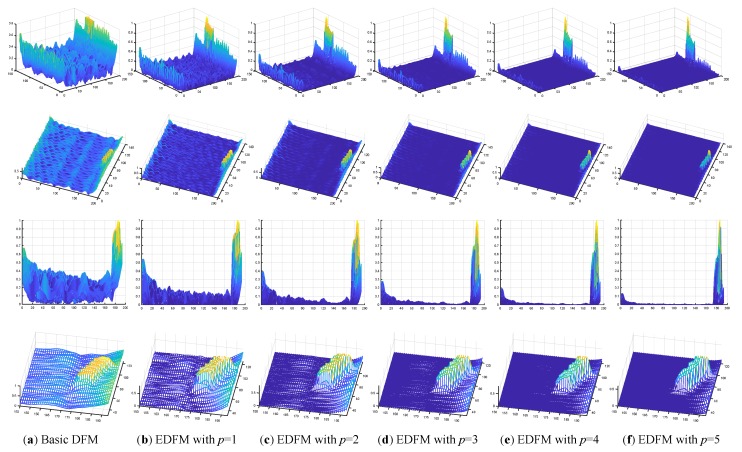
Exhibition of defect enhancement results with different background suppression index *p*. Rows 1–3 show 3D mesh surfaces of feature maps from different viewing angles, while Row 4 shows 3D mesh surfaces of defects in local regions.

As shown in Figure 14, we provide the comparative results of defect enhancement with different background suppression index *p*. Figure 14b–f presentes the enhanced feature maps of Figure 14a with the parameter p changing from 1 to 5. The background becomes smooth with the increase of parameter p, and the change can be clearly seen from in Row 3 at the side view. Moreover, the background noises shown in Row 2 are also suppressed to a certain extent, while the defect can be preserved and even enhanced. Row 4 of Figure 14 shows that the edge of defect becomes sharper with the increase of the background suppression index. Meanwhile, the discrimination between the defect and the background regions in EDFM becomes greater than in the basic DFM. In our work, the range of value for the background suppression index is always set to be from 3 to 5, which is determined by the complexity of background.

## 7. Conclusions

In this paper, a new analytical technology from the visualization perspective is presented to analyze the feature extraction method based on the GLCM, and on the basis of analyzing the changing regularity of co-occurrence gray values in GLCM, an effective texture descriptor with the LIDM feature distribution was proposed to characterize the tire X-ray texture image. Moreover, for the original tire texture image, the defective region makes up only a small portion of the whole image, and then the LIDM feature distribution calculated from the original tire image shows a similarity characteristic with that calculated from the non-defective image patches. Thus, through computing the Hausdorff distance between the LIDM feature distributions of the original image and each sliding patch, the DFM can be constructed to detect the defect conveniently. To improve the robustness and detection precision of our proposed defect detection method, we present an effective defect enhancement algorithm based on background suppression to enhance the DFM. Finally, we present the defect detection method to achieve the defect detection of tire X-ray image with a pixel-level precision. Experiments were conducted to validate the robustness and stability of our proposed defect detection method. As future work, we wish to improve the computing speed of LIDM feature distribution and investigate the defect recognition based on the pixel-level defect detection results.

## Figures and Tables

**Table 1:** Exhibition of Hausdorff distances between different LIDM feature distributions.

Distance	LIDM Features	$L\;I\;D\;M^{\chi_{a}}$	$L\;I\;D\;M^{\chi_{b}}$	$L\;I\;D\;M^{\chi_{c}}$	$L\;I\;D\;M^{\chi_{d}}$
LIDM Features					
$L\;I\;D\;M^{\chi_{a}}$		0	130.35	474.59	536.70
$L\;I\;D\;M^{\chi_{b}}$		130.35	0	494.25	545.76
$L\;I\;D\;M^{\chi_{c}}$		474.59	494.25	0	164.43
$L\;I\;D\;M^{\chi_{d}}$		536.70	545.76	164.43	0

**Table 2:** Detection time with different algorithms about typical X-ray images.

Detection Time (sec)	Image	Image 1	Image 2	Image 3	Image 4	Image 5	Image 6
Different Algorithms							
DFM + ACWE [36]		13.54	45.94	55.97	5.3	32.25	13.99
DFM + LBFACM [37]		45	52.18	19.03	9.75	21.87	18.57
DFM + TSWD		**1.12**	**0.81**	**0.77**	**0.35**	**0.74**	**1.39**
EDFM + ACWE [36]		13.95	45.61	54.5	5.21	7.88	13.32
EDFM + LBFACM [37]		17.01	3.24	8.31	3.56	11.2	14.17
EDFM + TSWD		**1.18**	**0.78**	**0.76**	**0.34**	**0.75**	**1.33**

**Table 3:** Comparative results of different methods.

Results	Performance Index	Image 1			Image 2			Image 3			Image 4			Image 5			Image 6		
Different Algorithms		*P* (%)	*R* (%)	*F* (%)	*P* (%)	*R* (%)	*F* (%)	*P* (%)	*R* (%)	*F* (%)	*P* (%)	*R* (%)	*F* (%)	*P* (%)	*R* (%)	*F* (%)	*P* (%)	*R* (%)	*F* (%)
DFM + ACWE [36]		39.8	100	56.9	3.20	100	6.30	6.80	100	12.7	82.1	94.0	87.8	18.9	100	31.8	18.9	100	31.8
DFM + LBFACM [37]		37.2	100	54.2	53.2	99.7	69.4	5.20	100	9.80	34.0	98.7	50.5	12.5	97.5	22.2	7.00	100	13.1
DFM + TSWD		51.8	100	68.2	69.0	95.8	80.2	53.5	100	69.7	48.7	100	65.5	57.8	96.0	72.2	58.2	100	73.4
EDFM + ACWE [36]		94.2	91.7	92.9	86.5	92.6	89.5	74.8	53.0	83.8	94.5	79.5	86.3	78.0	94.0	85.3	96.7	66.3	78.6
EDFM + LBFACM [37]		93.5	90.5	92.0	84.9	95.1	89.7	8.00	100	14.8	83.9	93.4	88.4	70.0	95.5	80.8	86.4	89.6	88.0
EDFM + TSWD		93.7	97.4	**95.5**	88.8	97.2	**92.8**	73.4	99.1	**84.3**	89.0	96.0	**92.4**	81.9	97.5	**89.0**	85.9	98.4	**91.7**

**Table 4:** Comparative results with state-of-the-art methods.

Results	Performance Index	Image 1			Image 2			Image 3			Image 4			Image 5			Image 6		
Different Algorithms		*P* (%)	*R*(%)	*F* (%)	*P* (%)	*R* (%)	*F* (%)	*P* (%)	*R* (%)	*F* (%)	*P* (%)	*R* (%)	*F* (%)	*P* (%)	*R* (%)	*F* (%)	*P* (%)	*R* (%)	*F* (%)
LSG [38]		46.4	100	63.4	40.3	94.7	56.6	24.7	82.4	38.0	34.5	97.5	51.0	76.4	92.1	83.5	42.6	100	59.8
Faster RCNN [25]		59.5	98.4	74.2	42.3	100	59.5	25.7	96.2	40.1	41.9	100	59.0	63.5	100	77.6	37.3	100	54.4
EDFM + TSWD		93.7	97.4	**95.5**	88.8	97.2	**92.8**	73.4	99.1	**84.3**	89.0	96.0	**92.4**	81.9	97.5	**89.0**	85.9	98.4	**91.7**
